# A Versatile Continuum Gripping Robot with a Concealable Gripper

**DOI:** 10.34133/cbsystems.0003

**Published:** 2023-02-24

**Authors:** Shuailong Zhang, Fenggang Li, Rongxin Fu, Hang Li, Suli Zou, Nan Ma, Shengyuan Qu, Jian Li

**Affiliations:** ^1^ School of Mechatronical Engineering, Beijing Institute of Technology, Beijing 100081, China.; ^2^ Beijing Advanced Innovation Center for Intelligent Robots and Systems, Beijing Institute of Technology, Beijing 100081, China.; ^3^ School of Medical Technology, Beijing Institute of Technology, Beijing 100081, China.; ^4^ School of Automation, Beijing Institute of Technology, Beijing 100081, China.; ^5^ Department of Mechanical, Materials, and Manufacturing Engineering, University of Nottingham, Nottingham, NG7 2QL, UK.

## Abstract

Continuum robots with their inherent compliance provide the potential for crossing narrow unstructured workspace and safely grasping various objects. However, the display gripper increases the size of the robots, and therefore, it tends to get stuck in constrained environments. This paper proposes a versatile continuum grasping robot (CGR) with a concealable gripper. The CGR can capture large objects with respect to the robot’s scale using the continuum manipulator and can grasp various objects using the end concealable gripper especially in narrow and unstructured workspaces. To perform the cooperative operation of the concealable gripper and the continuum manipulator, a global kinematic model based on screw theory and a motion planning approach referred to as “multi-node synergy method” for the CGR are presented. The simulation and experimental results show that objects of different shapes and sizes can be captured by the same CGR even in complex and narrow environments. Finally, in the future, the CGR is expected to serve for satellite capture in harsh space environments such as high vacuum, strong radiation, and extreme temperatures.

## Introduction

Working in a complex and tight space is challenging for both workers and rigid robots, especially gripping operations such as industrial pipe dredging, sampling for cave exploration, and satellite capture. The continuum robot is a kind of bionic robots whose continuum backbone can be continuously deformed [1–3]. The continuum robot does not contain rigid links and recognizable rotating joints, which are inspired by the soft bodies of creatures, such as elephant trunks, octopus arms, and snakes [4–10]. Compared with the discrete-jointed traditional rigid robots, the continuum robot has an increased self-adaptability and safe interaction capacity due to its inherent compliance [11–15]. Compared with the soft robots made of shape memory polymers [16] and conductive polymers [9], continuum robots have obvious advantages in robust grasps and efficient controllability. Continuum robots are ideal for these grasping tasks where the target workspaces are located far away from the robot’s access point and lot of obstacles exists between them [17–20].

**Fig. 1.**
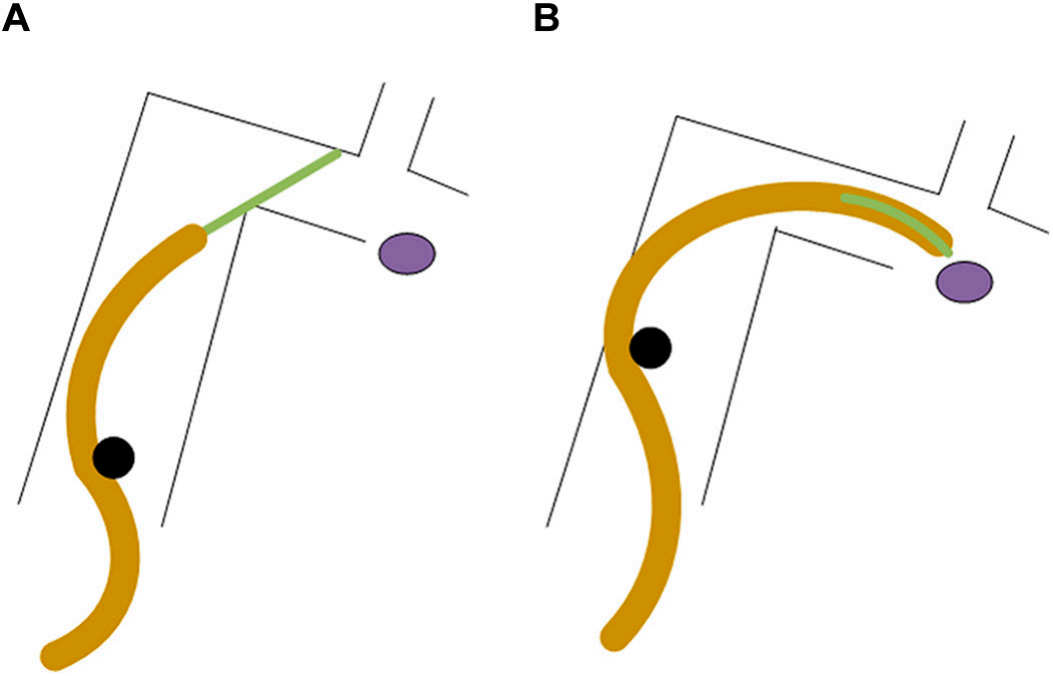
Illustration of a CGR with a concealable gripper that operates in a narrow tunnel environment, which performs better than the one with an exposed gripper. (A) A CGR with a rigid gripper tries to pass the tunnel and reach the target (purple) region but gets stuck at the corner of the tunnel. (B) CGR with a concealable gripper passes the tunnel and reaches the target region by hiding its last section.

How to improve the adaptability of the grasping robot to the shape and size of the target object and the adaptability to the complex environment has received the attention of many researchers. The relative published studies can be divided into 3 types. (a) Winding. The continuum grasping robots (CGRs) in this category include the Festo Bionic Motion Robot [21,22], inspired by an elephant trunk and octopus, as well as cellular pneumatic mesh manipulators [23]. They have good safe handling and adaptability, allowing easy grasping and movement of fragile or irregularly shaped objects, which is not the case for conventional rigid robots. However, these CGRs have difficulty in grasping small objects with respect to the robot’s scale because of the bending curvature limitations. (b) In combination with end effectors. Similar to traditional robots, the continuum robot can also grasp objects using end effectors. Yang et al. [24] installed a linear motor-driven gripper to a tendon-driven CGR for capturing small-sized objects. However, the drive of the gripper containing the rigid element is also located at the end of the CGR, which not only increases the load on the CGR but also limits its flexibility in narrow spaces. Alfalahi et al. [25] designed a concentric tube continuum robot with a flexible gripper at the end for intranasal skull base surgery. The robot gripper drive line is inserted through the innermost tube of the concentric tube manipulator, and the actuator is placed at the rear. This provides an idea for a lightweight design. Moreover, it is difficult for this concentric tube robot to capture objects of large size. The exposed gripers mentioned above are prone to getting stuck in a narrow position, and the forward direction could not be correctly selected by the passive compliance of the gripper during the forward process (Fig. 1A). On the other hand, the exposed grip increases the risk of entanglement in the environment, which can stop it from moving forward. (c) Multifinger collaboration. It simulates the human hand-grasping objects through multifinger coordination. The hydraulic CGR developed by Galloway et al. [26] allows flexible capture and sampling in the deep sea by controlling its internal pressure. CGRs of the same principle also include the humanoid soft hand [27] and the 6-finger gripper [28]. The presence of multiple fingers has good adaptability to objects of different sizes. However, a large size limits its use in a narrow space, and the load capacity of this kind of robot is limited. Therefore, it is necessary to develop a lightweight CGR that can capture objects of different sizes in narrow and complex spaces (pipe cleaning, space station garbage capture, and cave sampling). Moreover, a CGR with a concealable gripper provides a possibility for this (Fig. 1B).

In this paper, a CGR with a concealable gripper is proposed for grasping operations in complex and narrow spaces. Objects with various shapes and sizes can be captured by the cooperative operation of the concealable gripper or the continuum manipulator. The concealable gripper can be completely retracted into the backbone, which reduces the CGR volume when moving in tight spaces and avoids interference when the continuum manipulator performs coiling operations. When using only the rod-driven, the CGR has the potential to achieve a lightweight and compact design. Kinematics modeling and a motion planning approach, which is referred to as a “multi-node collaboration method,” are then introduced for the proposed robot. The novel motion planning method has advantageous characteristics. More precisely, it is a programming method in a global workspace, which does not require any convex assumption and does not depend on a convex index function of the searching space. In addition, allowing direct reference to the posture features of CGR makes the method user-friendly.

## Materials and Methods

### Design and analysis of CGR

**Fig. 2.**
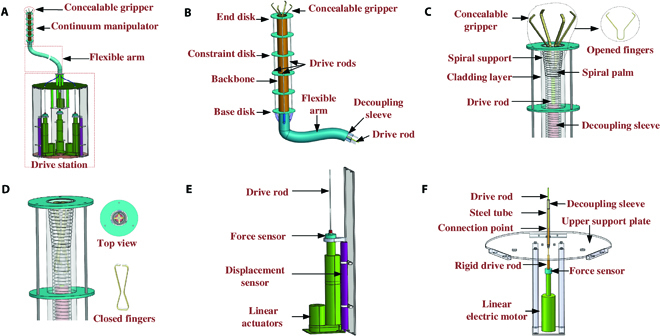
(A) Schematic of the CGR structure. (B) Schematic of a rod-driven continuum manipulator. (C and D) Schematic of the open and closed state of the rod-driven concealable gripper containing the opened and closed states of the fingers. (E) One of the linear drive modules for the continuum manipulator. (F) The linear drive module for the concealable gripper.

The proposed CGR contains 4 main parts (Fig. 2A). The end concealable gripper, the continuum manipulator, and the concealable gripper are connected as 1 unit, while the end concealable gripper and the continuum manipulator are connected to the drive station.

The continuum manipulator (Fig. 2B) is composed of a central backbone, 6 constrained disks, and 3 drive rods. The backbone, which cannot be stretched or compressed in the axial direction, allows bending and deflection movements. The 3 NiTi alloy rods for driving are rigidly connected at their tips to the end disc, and the part below the base disc is covered with a polyethylene decoupling sleeve.

The decoupling principle is similar to a bicycle brake system, where the deformation of the sleeve area does not change the length of the rod in the nonsleeve area. The flexible arm expands the CGR’s working range. The flexible arm allows the end effector to work in areas far from the drive station.

The 4 fingers of the concealable gripper are composed of 2 elastic sheets. The 2 elastic sheets are orthogonally arranged on the elastic palm by a gap fit (Fig. 2C). The open state of the concealable gripper can be elastically deformed into a closed state, under the constraint of the spiral support and the pull of the driving rod (Fig. 2D). The decoupling sleeve of the concealable gripper is fixed to the spiral support. In addition, the end of the spiral support is designed as a funnel, so that the elastic return force of elastic sheets helps the concealable gripper to unfold.

Three identical linear modules (Fig. 2E) are distributed on the rear drive station. The drive rod is always in tension under the antagonism of the pulling force of the driver and the restoring force of the elastic sheets. Displacement and force sensors are installed on the linear module to obtain the displacement and drive force of the drive rod.

The driver module of concealable gripper is shown in Fig. 2F. A rigid sleeve is installed outside the connection point of the rigid rod and the flexible rod. The rigid sleeve is rigidly connected to the upper support plate and the connection point does not move beyond the steel tube so that the thrust of the linear motor can be transmitted to the concealable gripper. The rigid rod is designed as a stepped shaft to restrain the upper limit of the actuator movement. The diameter of the elastic palm is larger than the inner diameter of the decoupling sleeve, which can restrain the lower limit of the linear movement.

**Fig. 3.**
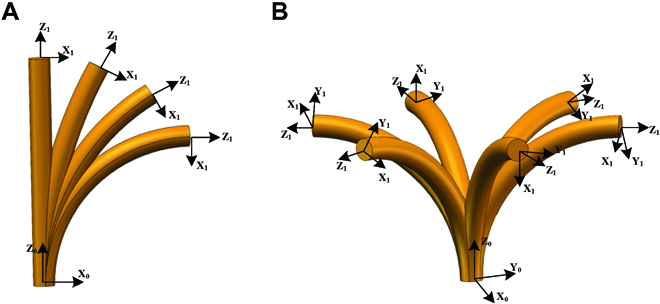
Schematic diagram of the bending and turnaround motion of the continuum manipulator. (A) The bending process of the continuum manipulator arm from 0° to 90°. (B) The turnaround process with twisting from 0° to 360°. The coordinate system at the end shows its end posture.

In addition, the following features should be mentioned. (a) The redundant actuated continuum manipulator allows complete bending (Fig. 3A) and winding (Fig. 3B) motion (2 degrees of freedom) by 3 drive rods. The inherent flexibility and suppleness of the continuum manipulator can reduce the impact force when coiling objects. (b) The concealable gripper is underactuated, and the opening and closing of its 4 fingers are achieved only by the axial movement of 1 driving rod. In addition, the concealable gripper can be retracted into the backbone, which reduces the size of the CGR and protects the flexible clamps. Small-sized objects can be grasped by the concealable gripper, while large objects with respect to the CGR’s scale can be captured by the continuum manipulator, which enriches the robot’s application scenarios. (c) The compliant flexible arm allows the CGR to work into narrow and unstructured spaces far from the drive station. (d) Compared with the drive mode of fluid and rotating joints, the rod-driven mode provides the potential for miniaturization and lightweight design of CGRs.

### Kinematics analysis of the CGR

#### Kinematics modeling of the continuum manipulator

**Fig. 4.**
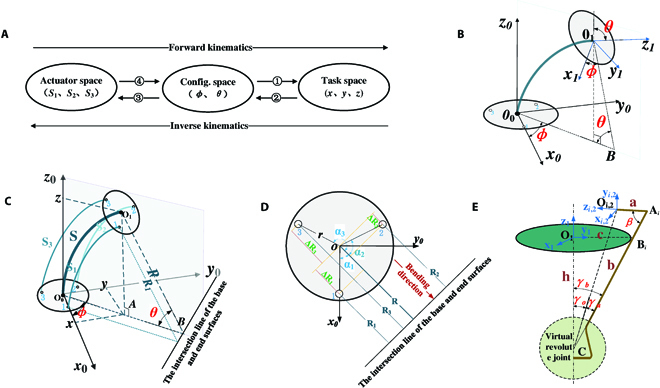
(A) The kinematic analysis process of the continuum manipulator. The complete forward kinematics includes ④ and ①, and the complete inverse kinematics includes ② and ③. Numbers ① to ④ express the order of introduction of the mapping. (B) The mapping of T and F can be obtained based on the model of the continuum manipulator workspace and configuration space. (C) The mapping of F and L can be obtained by the configuration space and model. (D) The corresponding end cross-sectional view of (C). (E) Kinematic model and parameter definition of a collet finger.

The configuration space and drive space of CGR are inconsistent because of the rod-driven method. Therefore, the kinematic parameters of the continuum manipulator arm are divided into 3 spaces (Fig. 4A). The driving space ***D*** = [*S*_1_ *S*_2_ *S*_3_]^T^ characterizes the length of the driving rods. The configuration space ***Θ*** = [*θ φ*]^T^ describes the shape of the manipulator, and the workspace ***W*** = [*x y z*]^T^ reflects the end position of the manipulator in Cartesian space. The complete forward kinematics includes ④ and ①, and the complete inverse kinematics includes ② and ③. Numbers ① to ④ express the order of introduction of the mapping.

The kinematic modeling of the continuum manipulator is performed based on the assumption of a constant curvature. In other words, the bending shape of the backbone is approximately an arc whose curvature is the same at all the positions during the movement [29,30].

A simplified motion model of the continuum manipulator is shown in Fig. 4B). ∑_0_(***o***_0_ − ***x***_0_***y***_0_***z***_0_) is the base frame, where ***O***_0_ is the center of the base disk. In addition, ∑_1_(***o***_1_ – ***x***_1_***y***_1_***z***_1_) is the end frame, which is fixed to the center ***O***_1_ of the base disk. Axes ***z***_0_ and ***z***_1_ are tangential to the backbone arc. Axes ***x***_0_ and ***x***_1_ are points to the first hole of the base disk and the end disk, respectively. The ***y*** axis can be determined by the right-hand rule. The dihedral angle θ∈[0,*π*] is defined as the angle between the base disk and end disk. The deflection angle *ϕ*∈[0,2*π*] is defined as the angle between the plane where the continuum manipulator is bent and the positive direction of the ***x***_0_ axis. Assuming that the length of the backbone’s arc can neither be compressed nor stretched, it is set to a fixed value ***s***.

1) **Mapping from configuration space to workspace**

When the continuum manipulator is working (*θ* ≠ 0), the position and posture of the end frame ∑_1_ can be obtained by 4 basic transformations from ∑_0_. The base frame ∑_0_ is first translated from ***O***_0_ to ***O***_1_ to obtain frame ∑_0,1_. The latter is then rotated around its ***z*** axis by an angle *ϕ* to obtain frame ∑_0,2_. Afterward, frame ∑_0,2_ is rotated around its ***y*** axis by an angle *θ* to obtain frame ∑_0,3_. Finally, frame ∑_0,3_ is rotated around its ***z*** axis by an angle −*ϕ* to obtain frame ∑_1_. The twist coordinates of the 4 decomposed motions can be expressed as:

$$\begin{array}{c} \xi_{1}=\mu {\left[(1-\cos\theta )\cos\phi (1-\cos\theta )\sin\phi \;\sin\theta \;0\;0\;0\right]}^{T}\\ \xi_{2}={\left[\begin{array}{cccccc} 0 & 0 & 0 & 0 & 0 & 1 \end{array}\right]}^{T}\\ \xi_{3}={\left[\begin{array}{cccccc} 0 & 0 & 0 & 0 & 1 & 0 \end{array}\right]}^{T}\\ \xi_{4}={\left[\begin{array}{cccccc} 0 & 0 & 0 & 0 & 0 & 1 \end{array}\right]}^{T} \end{array}$$ (1)

where $u=1/\sqrt{2(1-\text{cos}\theta_{i})}$.

The twists of the twist coordinates are given by:

$$\begin{array}{c} {\hat{\xi }}_{1}=u[\begin{array}{cccc} 0 & 0 & 0 & (1-\cos\theta )\cos\phi \\ 0 & 0 & 0 & (1-\cos\theta )\sin\phi \\ 0 & 0 & 0 & \sin\theta \\ 0 & 0 & 0 & 0 \end{array}],{\hat{\xi }}_{2}=[\begin{array}{cccc} 0 & -1 & 0 & 0\\ 1 & 0 & 0 & 0\\ 0 & 0 & 0 & 0\\ 0 & 0 & 0 & 0 \end{array}],\\ {\hat{\xi }}_{3}=[\begin{array}{cccc} 0 & 0 & 1 & 0\\ 0 & 0 & 0 & 0\\ -1 & 0 & 0 & 0\\ 0 & 0 & 0 & 0 \end{array}],{\hat{\xi }}_{4}=[\begin{array}{cccc} 0 & -1 & 0 & 0\\ 1 & 0 & 0 & 0\\ 0 & 0 & 0 & 0\\ 0 & 0 & 0 & 0 \end{array}] \end{array}$$ (2)

where the symbol ^ is the wedge operator [31], which maps a vector from ℜ^6^ to the Lie algebra ***se***(3).

By applying the product of the exponentials formula [32], the transformation from the base frame ∑_0_ to the end frame ∑_1_ can be expressed as:

$$\begin{array}{c} T_{1}^{0}=\exp({\hat{\xi }}_{1}L_{1})\text{exp}({\hat{\xi }}_{2}\varphi )\text{exp}({\hat{\xi }}_{3}\theta )\text{exp}(-{\hat{\xi }}_{4}\varphi )\\ =[\begin{array}{cc} R_{1}^{0} & P_{1}^{0}\\ 0 & 1 \end{array}] \end{array}$$ (3)

where  $L_{1}=S\sqrt{2(1-\text{cos}\theta )}/\theta$, $R_{1}^{0}$, and $P_{1}^{0}$ can be computed as:

$$R_{1}^{0}=[\begin{array}{ccc} {\text{cos}}^{2}\varphi \text{cos}\theta +{\text{sin}}^{2}\varphi & \text{cos}\varphi \text{sin}\varphi (\text{cos}\theta -1) & \text{cos}\varphi \text{sin}\theta \\ \text{cos}\varphi \text{sin}\varphi (\text{cos}\theta -1) & {\text{sin}}^{2}\varphi \text{cos}\theta +{\text{cos}}^{2}\varphi & \text{sin}\varphi \text{sin}\theta \\ -\text{cos}\varphi \text{sin}\theta & -\text{sin}\varphi \text{sin}\theta & \text{cos}\theta \end{array}]$$

$$P_{1}^{0}=\frac{S}{\theta }{\left[\begin{array}{ccc} (1-\text{cos}\theta )\text{cos}\varphi & (1-\text{cos}\theta )\text{sin}\varphi & \text{sin}\theta \end{array}\right]}^{T}$$

2) **Mapping from joint space to drive space**

This section details the mapping from ***Θ*** = [*θ φ*]^T^ to ***D*** = [*S*_1_ *S*_2_ *S*_3_]^T^ of the continuum manipulator. The geometric relationship between ***Θ*** and ***D*** is shown in Fig. 4C). Figure 4D presents the cross-sectional view of the ***x***_0_-***o***_0_-***y***_0_ of Fig. 4C.

It can be deduced from Fig. 4D that:

$$\alpha_{1}=\phi ,\;\alpha_{2}=120{}^{\circ}-\phi ,\;\alpha_{3}=240{}^{\circ}-\phi$$ (4)

where *α_i_*(*i* = 1,2,3) is the angle between vector $\vec{r_{i}}$ (vector from the center O_0_ to the center of the *i*th hole) and the plane where the backbone is bent. The radius of curvature *R_i_* corresponding to the drive rod can be expressed as:

$$R_{i}=S/\theta -r\text{cos}\alpha_{i}\;\;(\theta \neq 0)$$ (5)

The length of the drive rods is then given by:

$$S_{i}=\theta \times R_{i}\;\;i=1,2,3$$ (6)

Therefore, the mapping from joint space to drive space for the continuum manipulator can be expressed as:

$$\left\{ \begin{array}{c} S_{1}=S-\mathrm{\theta r}\text{cos}\phi \\ S_{2}=S-\mathrm{\theta r}\text{cos}(2\pi /3-\phi )\\ S_{3}=S-\mathrm{\theta r}\text{cos}(4\pi /3-\phi ) \end{array}\right.$$ (7)

3) **Mapping from drive space to joint space**

It can be deduced from Eq. 7 that:

$$\left\{ \begin{array}{c} S_{1}+\mathrm{\theta r}\cos\phi =S_{2}+\mathrm{\theta r}\text{cos}(2\pi /3-\phi )\\ S_{1}+\mathrm{\theta r}\text{cos}\phi =S_{3}+\mathrm{\theta r}\text{cos}(4\pi /3-\phi ) \end{array}\right.$$ (8)

Solving the above equations can lead to the mapping from the driving space to the joint space:

$$\left\{ \begin{array}{c} \phi =\text{arctan}\frac{\sqrt{3}(S_{2}-S_{3})}{2S_{1}-S_{2}-S_{3}}\\ \theta =\frac{S_{2}-S_{1}}{r(3\text{cos}\phi -\sqrt{3}\text{sin}\phi )} \end{array}\right.$$ (9)

4) **Mapping from workspace to joint space**

Considering the range of *ϕ*_∈_[0,2*π*], *ϕ* can be expressed by Eq. 11 according to the geometric relationship presented in Fig. 4C:

$$\phi =\{\begin{array}{cc} \text{arccos}(\frac{x_{1}}{\sqrt{{x_{1}}^{2}+{y_{1}}^{2}}}) & y_{1}\geq 0\\ 2\pi -\text{arccos}(\frac{x_{1}}{\sqrt{{x_{1}}^{2}+{y_{1}}^{2}}}) & y_{1}<0 \end{array}$$ (10)

The bending angle can be easily obtained from Fig. 4C:

$$\theta =2\arcsin\sqrt{\frac{{x_{1}}^{2}+{y_{1}}^{2}}{{x_{1}}^{2}+{y_{1}}^{2}+{z_{1}}^{2}}}$$ (11)

Therefore, the mapping from the workspace to the joint space can be expressed by Eqs. 10 and 11.

#### Kinematics modeling of concealable gripper

The underactuated concealable gripper consists of 4 collet fingers. It is assumed that the movement of each collet finger is equivalent to rotating around a virtual joint. The coupling deformation of the concealable gripper and the continuum manipulator is ignored. In addition, the length of the collet finger ***A***_***i***_***C*** is considered as a fixed value (b), as shown in Fig. 4E). ***B***_***i***_ is the contact point between the inner wall of the spiral support and the *i*th collet finger. The frame ∑_***i***,2_(***o***_***i***,2_ − ***x***_***i***,2_***y***_***i***,2_***z***_***i***,2_)(***i***=1,2,3,4) is fixed to the end point ***O***_***i***,2_ of the ***i***th collet finger. Axes ***z***_***i***,2_ and ***A***_***i***_***O***_***i***,2_ are collinear, while axes ***x***_***i***,2_ and ***x***_1_ are parallel.

1) **Mapping between drive space and joint space**

The length ***h***, which is the driving space parameter, is defined as the distance between the end disc center *O*_1_ and virtual joint center ***C***. When the concealable gripper is just completely closed, ***h*** is written as ***h***_0_. The boundary angle *γ*_***b***_ is defined as the rotation angle from the boundary ***CA***_***i***_ of the ***i***th collet finger to the reference axis ***CO***_1_. *γ*_o_ is the opening angle between ***CO***_1_ and ***CO***_***i***,2_. *γ*_u_ is the underlying angle between ***CO***_***i***,2_ and ***CA***_***i***_. The mapping of the drive space to the joint space is given by:

$$\gamma_{b}=\arctan(\frac{c}{h})$$ (12)

where length ***c*** is the diameter of the inner wall of the spiral support.

Furthermore, the mapping from joint space to drive space can be obtained:

$$\Delta h=\frac{c}{\tan\gamma_{b}}-h_{0}$$ (13)

where the change value Δ*h* represents the pulling length of the drive rod, and the initial setting $h_{0}=\sqrt{b^{2}-c^{2}}$ is the length of the drive rod when the concealable gripper is just completely closed.

The opening angle of the concealable gripper is expressed as:

$$\gamma_{o}=\text{arctan}(\frac{c}{h})-\text{arctan}(\frac{a\;\text{sin}\beta }{b-a\;\text{cos}\beta })$$ (14)

2) **Mapping between joint space and workspace**

The position and posture of the end frame ∑_***i***,2_ of the ***i***th collet finger can be obtained by 5 basic transformations from frame ∑_1_. (a) Frame ∑_1_ is first translated from ***O***_1_ to ***C*** to obtain frame ∑_1,1_. (b) In order to obtain the point ***A***_***i***_ on different fingers, frame ∑_1,1_ is rotated around its ***x*** axis by finger angle *η*(*η* = i*π/2) to obtain frame ∑_***i***,1,2_. (c) Afterward, frame ∑_***i***,1,2_ is translated from ***C*** to ***A***_***i***_ to obtain frame ∑_***i***,1,3_. (d) Frame ∑_***i***,1,3_ is then rotated around its ***x*** axis by angle *β*′(*β*′ = π-*β*−*γ*_b_) to obtain frame ∑_***i***,1,4_. (e) Finally, frame∑_***i***,1,4_ is translated from ***A***_***i***_ to ***O***_***i***,2_ to obtain frame ∑_***i***,2_. The corresponding twist coordinates of the 5 decomposed motions can be expressed as:

$$\left\{ \begin{array}{c} \xi_{5}={\left[\begin{array}{cccccc} 0 & 0 & 1 & 0 & 0 & 0 \end{array}\right]}^{T}\\ \xi_{6}={\left[\begin{array}{cccccc} 0 & 0 & 0 & 0 & 0 & 1 \end{array}\right]}^{T}\\ \xi_{7}={\left[\begin{array}{cccccc} 0 & \text{sin}\gamma_{b} & \text{cos}\gamma_{b} & 0 & 0 & 0 \end{array}\right]}^{T}\\ \xi_{8}={\left[\begin{array}{cccccc} 0 & 0 & 0 & 1 & 0 & 0 \end{array}\right]}^{T}\\ \xi_{9}={\left[\begin{array}{cccccc} 0 & 0 & 1 & 0 & 0 & 0 \end{array}\right]}^{T} \end{array}\right.$$ (15)

The transformation from the base frame ∑_1_ to the end frame ∑_***i***,2_ is given by:

$$\begin{array}{c} T_{i,2}^{1}=\text{exp}(-{\hat{\xi }}_{5}h)\text{exp}({\hat{\xi }}_{6}\eta )\text{exp}({\hat{\xi }}_{7}b)\text{exp}({\hat{\xi }}_{8}\beta^{'})\text{exp}({\hat{\xi }}_{9}a)\\ =[\begin{array}{cc} R_{i,2}^{1} & P_{i,2}^{1}\\ 0 & 1 \end{array}] \end{array}$$ (16)

where $h=\frac{c}{\tan\gamma_{b}}$ is the distance from ***O***_1_ to ***C***.

For the ***i***th collet finger, the forward kinematics can be written as:

$$T_{i,2}^{0}=T_{1}^{0}T_{i,2}^{1}$$ (17)

The pose of the center of the 4 fingers in frame ∑_1_ can be expressed as:

$$T_{2}^{1}=[\begin{array}{cc} I_{3\times 3} & P_{2}^{1}\\ 0 & 1 \end{array}]$$ (18)

where I_3×3_ ∈ ℜ_3_ is the identity matrix. Element $P_{i,2}^{1}(3)$ in $P_{2}^{1}={\left(\begin{array}{ccc} 0 & 0 & P_{i,2}^{1}(3) \end{array}\right]}^{T}$ is similar to the third one of $P_{i,2}^{1}$. The pose of the center of the 4 fingers is given by:

$$T_{2}^{0}=T_{1}^{0}T_{2}^{1}$$ (19)

### Motion planning of the CGR

**Fig. 5.**
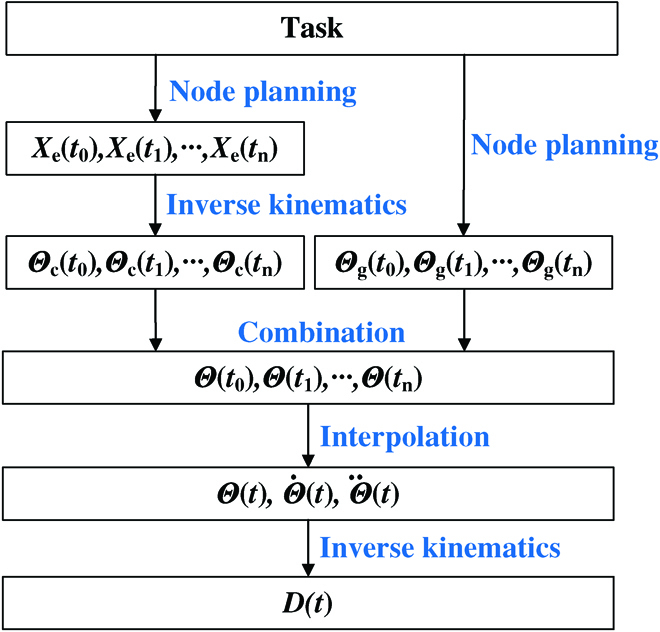
Process of the multi-node synergy method of the CGR. The nodes *X_c_* of the continuum manipulator in the workspace and the nodes *θ_g_* of the hidden gripper in the configuration space are planned according to the task. The configuration space node parameters *θ_c_* of the continuum manipulator are obtained through the inverse kinematics solution. The configuration space parameters *θ* of the nodes are composed of the combination of *θ_c_* and *θ_g_* . Finally, the drive parameters *D* for drive control are obtained by interpolation and inverse kinematics solution.

The CGR allows the gripping and handling of objects, which usually requires the coordinated operation of the continuum manipulator and the concealable gripper. To this end, a novel motion planning approach, referred to as the “multi-node synergy method” for the CGR is proposed (Fig. 5). In this method, the key path nodes for the concealable gripper are first planned in Cartesian space, according to the task requirements. The parameters of the key path nodes contain the positions *X*_e_(*t*_0_),*X*_e_(*t*_1_),⋯,*X*_e_(*t_n_*) of the grip points (i.e., centers of the 4 finger tips of the concealable gripper) and the boundary angles *Θ*_g_(*t*_0_),*Θ*_g_(*t*_1_),⋯,*Θ*_g_(*t_n_*). The joint space node *Θ*_c_(*t*_0_),*Θ*_c_(*t*_1_),⋯,*Θ*_c_(*t_n_*) for the continuum manipulator can then be obtained by inverse kinematics. Afterward, the complete joint space node *Θ*(*t*_0_),*Θ*(*t*_1_),⋯,*Θ*(*t_n_*) for the CGR, combined with the joint space nodes for the continuum manipulator and the boundary angles, can be expressed as Θ = [Θ_c_ Θ_g_]. The joint angle *Θ*(*t*), angular velocity $\dot{\Theta }\left(t\right)$, and angular acceleration $\ddot{\Theta }\left(t\right)$ are then obtained according to the interpolation function. Finally, the drive space trajectory planning *D*(*t*) for CGR is performed by the inverse kinematics from the joint space to the drive space.

## Results

### Numerical simulation

The CGR is numerically simulated and analyzed based on the designed prototype and the proposed kinematics algorithm. The parameters used in the simulations are presented in Table 1.

**Table 1.** Structure parameters of the CGR.

Parameter	Symbol	Value	Unit
Length of backbone	S	200	mm
Dihedral angle	*θ*	[0,*π*]	rad
Deflection angle	*ϕ*	[0,2*π*]	rad
Distance from the rod holes to the center of constraining disk	r	**25**	mm
Length of the collet fingertip	a	**5**	mm
Length of the collet finger	b	**30**	mm
Diameter of the spiral support	c	**12**	mm
Boundary angle	*γ*_***b***_	$[\frac{29}{360}\frac{149}{360}]\pi \;\text{rad}$	rad

#### Workspace analysis

**Fig. 6.**
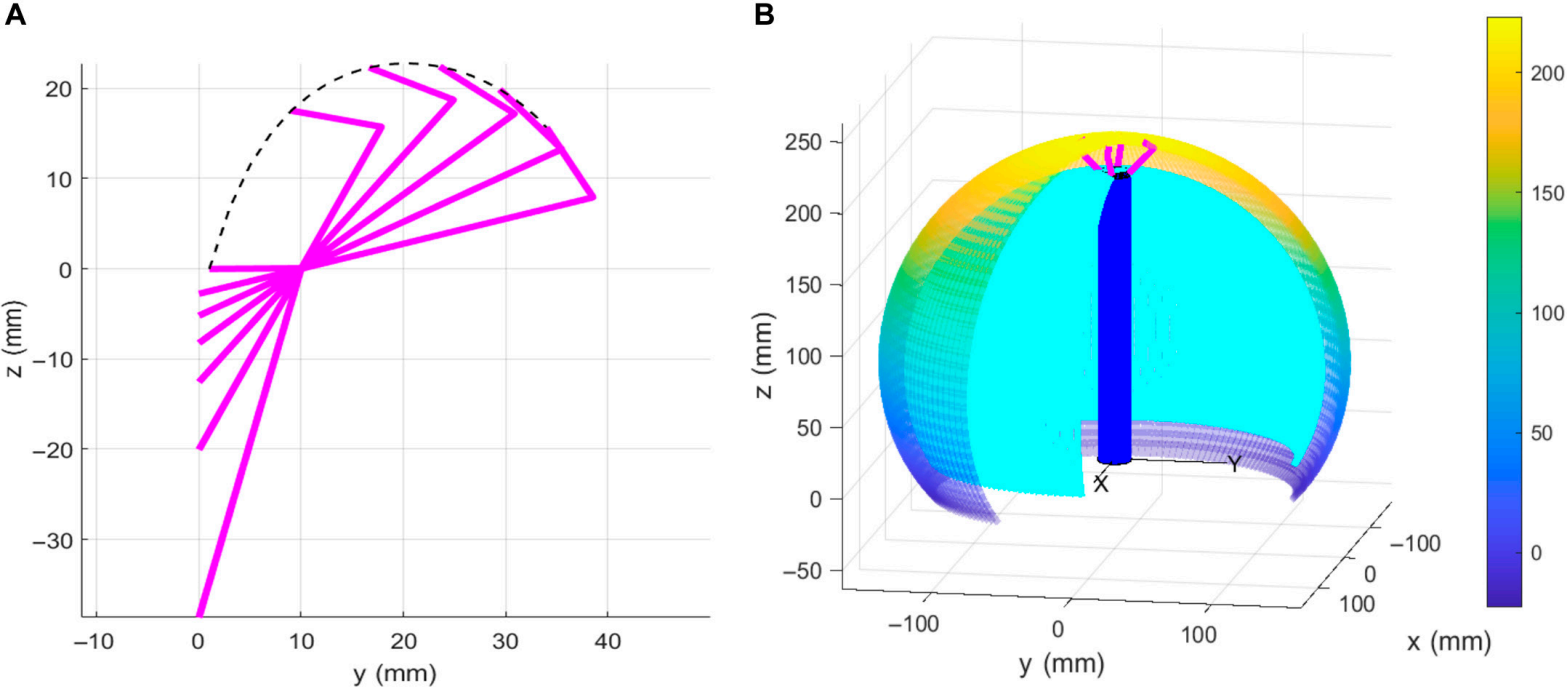
(A) Motion path of the first collect finger of the GCR. (B) Sectional view of the co-working space of the continuum manipulator and concealable gripper.

The reachable workspace is an important basis for judging the performance of GCR. The path of the first collect finger is shown in Fig. 6A according to the positive kinematic Eq. 16 and the parameter variation range in Table 1. The extension length of the concealable gripper along the *z* axis is the largest (22.79 mm) when the boundary angle is *γ*_***b***_ = 0.77. The maximum open radius (33.98 mm) of the concealable gripper is taken when the boundary angle is *γ*_***b***_ = 1.30.

The co-working space of the continuum manipulator and concealable gripper is shown in Fig. 6B. Note that the cooperative workspace is an eggshell-shaped envelope area. The cyan eggshell surface is the reachable space for the tip of the continuum manipulator, while the area between the colored eggshell surface and the cyan eggshell surface is the reachable space for the center of the end concealable gripper. The limit range of the concealable gripper is *x*_max_ = *y*_max_ = 162.98 mm, *x*_min_ = *y*_min_ = −162.98 mm, *z*_max_ = 222.80 mm, and *z*_min_ = −22.79 mm. The workspace limit range of the continuum manipulator is *x*_max_ = *y*_max_ = 144.90 mm, *x*_min_ = *y*_min_ = −144.90 mm, *z*_max_ = 200 mm, and *z*_min_ = 0 mm.

#### Simulation of cooperative motion

Moving the target object is one of the common operations of the grasping robot. To verify the efficiency of the kinematics model, a numerical simulation analysis is performed on the grasping and moving tasks. Assuming that there is a static spherical target object having a radius of 20 mm in the reachable workspace, it is required to move the center of the sphere from point A [98 98 127]^T^ (mm) to point B [70 120 127]_T_ (mm) by the CGR.

The node parameters for this motion planning are presented in Table 2. The angular velocity and angular acceleration parameters for each node are set to 0.

**Table 2.** Multi-node linkage trajectory planning parameters of the CGR.

Node	θ/°	φ/°	γ/°	Time (t/s)
0	0	45	14.5	0
1	90	45	70	15
2	90	45	25	25
3	90	120	25	35
4	90	120	70	45
5	0	45	14.5	60

**Fig. 7.**
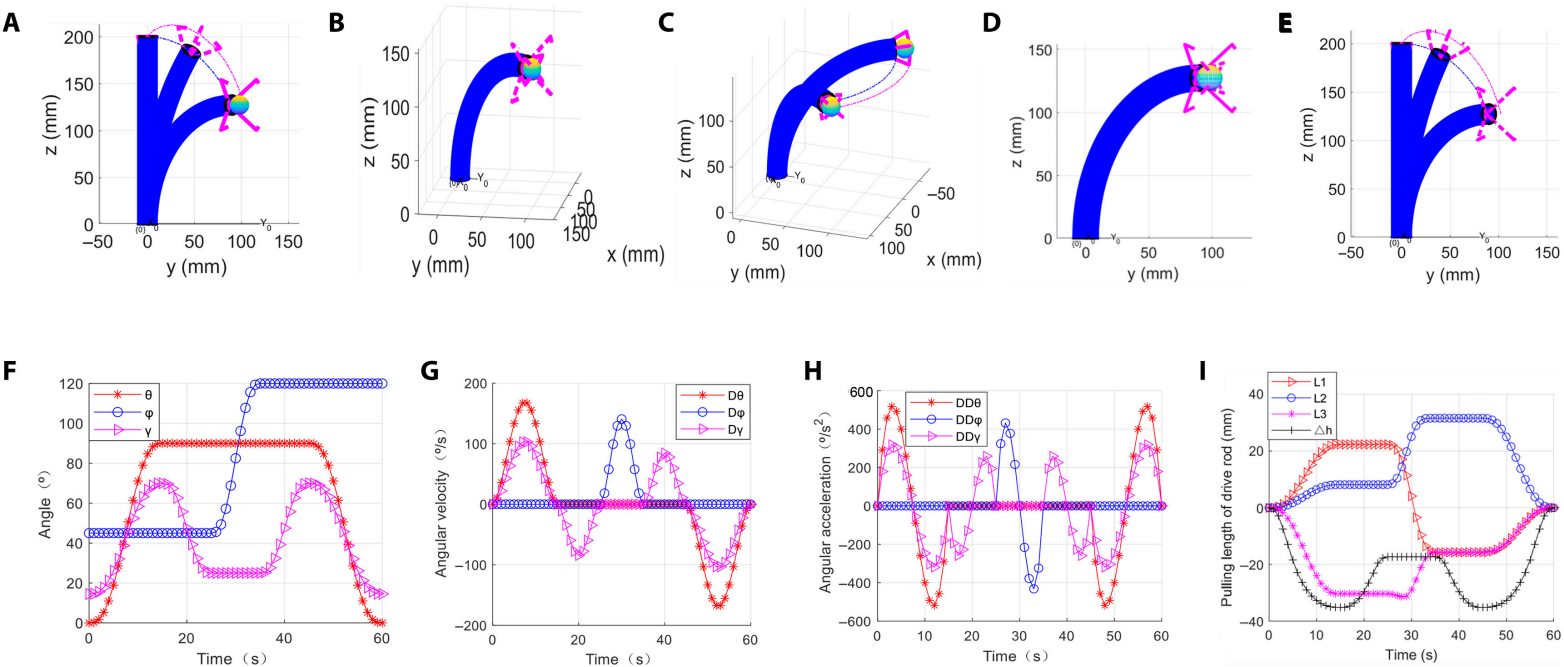
Simulation of transport tasks including approach (A), grab (B), handle (C), release (D), and reset (E) operations. (F to H) The configuration space parameters in the transfer task including angle (F), angular velocity (G), and angular acceleration (H). Drive space parameters corresponding to the transport task.

The CGR achieves the above tasks through 5 basic actions (Fig. 7A to E): (a) Approaching the target. When approaching a target object, the concealable gripper should be opened at the same time in a coordinated motion. (b) Gripping the target. After reaching point A, the continuum manipulator remains stationary while the concealable gripper completes the gripping action independently. (c) Moving the target. The concealable gripper maintains its grip on the target objects, and the continuum manipulator moves them from point A to point B. (d) Releasing the target. This is the inverse process of grabbing the target. (e) Resetting. This is the inverse process of approaching the target.

The angles, angular velocities, and angular accelerations of the continuum manipulator when performing transport task based on a 5-polynomial interpolation are shown in Fig. 7F to H). Moreover, the drive space results corresponding to the transport task are shown in Fig. 7I. The length of each drive rod changes continuously and smoothly throughout the operation, which can efficiently avoid the drive damage caused by sudden speed changes.

### Experiment

#### Experiment of cooperative motion

**Fig. 8.**
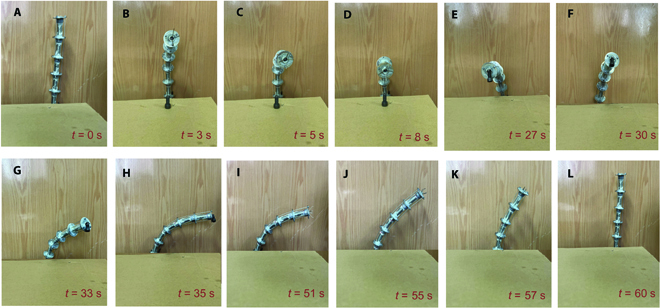
The experimental verification corresponding to the simulation. The bolt located on the table is successfully moved away from the table. The process of the CGR approaching bolt (A to D), transferring bolt (E to H), and restoring (I and J). The process of grabbing and releasing the bolt is similar to that shown in the Supplementary Materials and will not be repeated here.

The driving space parameters in Fig. 7D are used as the driving input of the prototype, and the bolt located on the table is successfully moved away from the table (Fig. 8). The kinematic model and motion planning method proposed in this paper are reasonable, which is proved by the consistency between the above experimental results and simulation results.

#### Experiment of application

This section first illustrate the adaptability of CGR using the concealable gripper to pinch various styles of objects and grasp the same object in multiple ways. Then, the continuum manipulator is used to wrap objects with a larger size, which are not easy to be pinched by the concealable gripper, to demonstrate the complementarity of the manipulator arm and the hidden gripper in the grasping operation. Finally, the CGR performs grabbing tasks in a simulated constrained environment, demonstrating its potential for applications in cave sampling, pipeline cleaning, and space station maintenance.

**Fig. 9.**
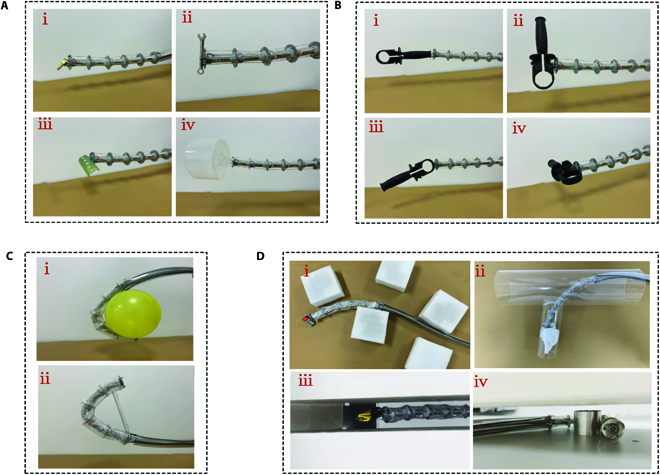
Experimental image of CGR operations. (A) The CGR pinches a (i) point, (ii) line, (iii) surface, and (iv) body-shaped object by the concealable gripper. (B) The concealable gripper pinches the same unstructured object through the (i) tail, (ii) waist, (iii) head, and (iv) lateral waist. (C) Objects with larger dimensions, such as balloons (i) and iron plates (ii), are grabbed by the continuum manipulator. (D) The CGR operates in simulated constrained environments. (D) (i) A bolt deep in the unstructured space is captured by concealable gripper. (ii) The blockage of the pipe is cleared by the CGR. (iii and iv) Samples in cramped environments are obtained by pinching and wrapping.

Objects of different shapes and sizes could be pinched by the concealable gripper. The block bolts, linear wrenches, flat sheet metal, and three dimensional (3D) shaped disc storage buckets could be stably pinched by the concealable gripper (Fig. 9A). It can be deduced that the volume is not the decisive factor affecting the grasping, and objects with larger volumes can also be grasped as long as there are suitable points for grasping. In addition, the same object could be pinched in different ways. The plastic handle can be stably held by grabbing (Fig. 9B) the (i) tail, (ii) waist, (iii) head, and (iv) lateral waist using the concealable gripper. It is crucial for the target object grasping in uncertain scenes.

The large-sized objects with respect to the CGR’s scale could also be wrapped with a continuum manipulator. The inherent flexibility of the backbone allowed the continuum manipulator to conform to balloons that could not be grasped by the concealable gripper upon contact (Fig. 9C, i). This does not imply that the grasped target object necessarily passes through its fitting continuum manipulator. The edge of the target object in contact with the continuum manipulator may also be grasped as long as the force closure condition is satisfied (Fig. 9C, ii). The continuum manipulator and the concealable gripper have complementary advantages when grasping a variety of target objects.

Grasping objects in complex and confined environments is particularly attractive through the CGR. Bolts in complex obstacles (Fig. 9D, i) and towels blocking pipes (Fig. 9D, ii) could be pinched by the concealable gripper of the CGR, assisted by the continuous deformation of the continuum manipulator. The concealable gripper can be completely retracted into the protective backbone, which can help the CGR flexibly move through the narrow and complicated space, and has a protective effect on the concealable gripper. In addition, knives dropped in narrow wall gaps, retrieved by the concealable gripper (Fig. 9D, iii), and hoops under the sofa could be captured by the continuum manipulator (Fig. 9D, iv). Finally, potential applications for CGR’s operations include post-disaster rescue, pipe cleaning, and cave sampling.

## Conclusion

In this study, a CGR with a concealable gripper is first proposed for grasping operations in complex and narrow spaces. A global kinematic model based on the screw theory and a motion planning approach referred to as “multi-node synergy method” for the CGR are then presented to perform the cooperative operation of the concealable gripper and the continuum manipulator. In addition, using rods to transmit power from the rear, the CGR has the potential to achieve a lightweight and compact design, and the fragile control system can be safely protected at the rear.

In future work, we aim at combining the adaptability of changing scenarios, the safety of interaction, and a higher load capacity, which is challenging and crucial for for flexible grasping, especially for the capture of space junk, pipeline maintenance, and cave exploration. In addition, accurately obtaining the grasping force exerted by the continuum robot on the target object and the contact force when the robot body interacts with the environment,especially in narrow and complex environments, is of our interest.

## Data Availability

The data used to support the findings of this study are included within the article.
